# Supplementary material for: Striping of orbital-order with charge-disorder in optimally doped manganites
Source: Nat Commun. 2021 Nov 3;12:6319. doi: 10.1038/s41467-021-26625-w (PMC8566459; doi:10.1038/s41467-021-26625-w)
Supplement: Supplementary file 1 — Supplementary information. [file 41467_2021_26625_MOESM1_ESM.pdf]

Supporting information for: Striping of orbital-order with charge-disorder in optimally doped manganites

Wei-Tin Chen[1,2,*], Chin-Wei Wang[3], Ching-Chia Cheng[1], Yu-Chun Chuang[3], Arkadiy Simonov[4], Nicholas C. Bristowe[5], Mark S. Senn[6,†]

[1]Center for Condensed Matter Sciences, National Taiwan University, Taipei 10617, Taiwan
[2]Taiwan Consortium of Emergent Crystalline Materials, Ministry of Science and Technology, Taipei 10622, Taiwan
[3]National Synchrotron Radiation Research Center, Hsinchu 30076, Taiwan
[4]Materials Department, ETH Zürich, Vladimir-Prelog-Weg 1-5/10, 8093 Zürich, Switzerland
[5]Centre for Materials Physics, Durham University, South Road, Durham, DH1 3LE, United Kingdom
[6]Department of Chemistry, University of Warwick, Gibbet Hill, Coventry, CV4 7AL, United Kingdom

*weitinchen@ntu.edu.tw
†m.senn@warwick.ac.uk

A =	GoF	a (Å)	e0 (%)	Mn_A^{BVS}	Mn_B^{BVS}	B-O-B (°)	2nd Phase %	Δ(a) %
Na	1.41	7.335350(8)	0.012	2.953(6)	3.560(3)	139.76(6)	36.6	-0.05
$Na_{0.9}$	1.15	7.337897(8)	0.022	2.987(6)	3.535(3)	139.44(6)	10.7	0.048
$Na_{0.8}$	1.37	7.342502(6)	0.027	2.962(5)	3.530(3)	139.55(5)	42.9	0.025
$Na_{0.7}$	1.19	7.351718(6)	0.013	2.917(5)	3.515(3)	139.69(5)	41.7	-0.05
$Na_{0.6}$	1.12	7.358369(7)	0.016	2.937(5)	3.478(3)	139.35(5)	31.1	-0.047
$Na_{0.5}$	1.33	7.365460(5)	0.009	2.963(5)	3.439(3)	138.99(5)	16.7	-0.027
$Na_{0.4}$	1.08	7.371698(5)	0.010	2.930(5)	3.429(3)	139.10(5)	4.7	-0.089
$Na_{0.3}$	1.39	7.37455(1)	0.033	2.937(7)	3.415(3)	138.97(6)	33.5	0.039
$Na_{0.2}$	1.52	7.384331(5)	0.007	2.965(7)	3.365(3)	138.54(6)	11.6	-0.01
$La_{0.2}$	1.13	7.41556(1)	0.015	2.919(7)	3.280(3)	138.35(6)	27.0	0.024
$La_{0.4}$	1.29	7.436686(8)	0.014	2.905(6)	3.203(3)	137.88(6)	31.61	0.014
$La_{0.6}$	1.19	7.45605(1)	0.046	2.910(6)	3.139(3)	137.55(5)	30.1	0.027
$La_{0.8}$	1.33	7.47914(1)	0.035	2.838(6)	3.095(3)	137.68(5)	15.2	-0.033
La	1.52	7.496075(6)	0.007	2.817(9)	3.049(4)	137.54(8)	12.0	-0.015

Table S1: Details of refinements of AMn_7O_{12} against ID22 data at 700 K. Refined structures are deposited in CIF format as part of the electronic SI. A = $Na_{0.1}Ca_{0.9}$ was not measured at 700K and A = Ca had extreme preferred orientation and so no reliable refinement was possible at all temperature. A= La has anti-site order that is modeled by a size broadening model for *h,k,l* = 2n subset of reflections. Representative Rietveld fits are given in Fig. S1. Crystallographic information files are included as part of the electronic Supplemental Information.

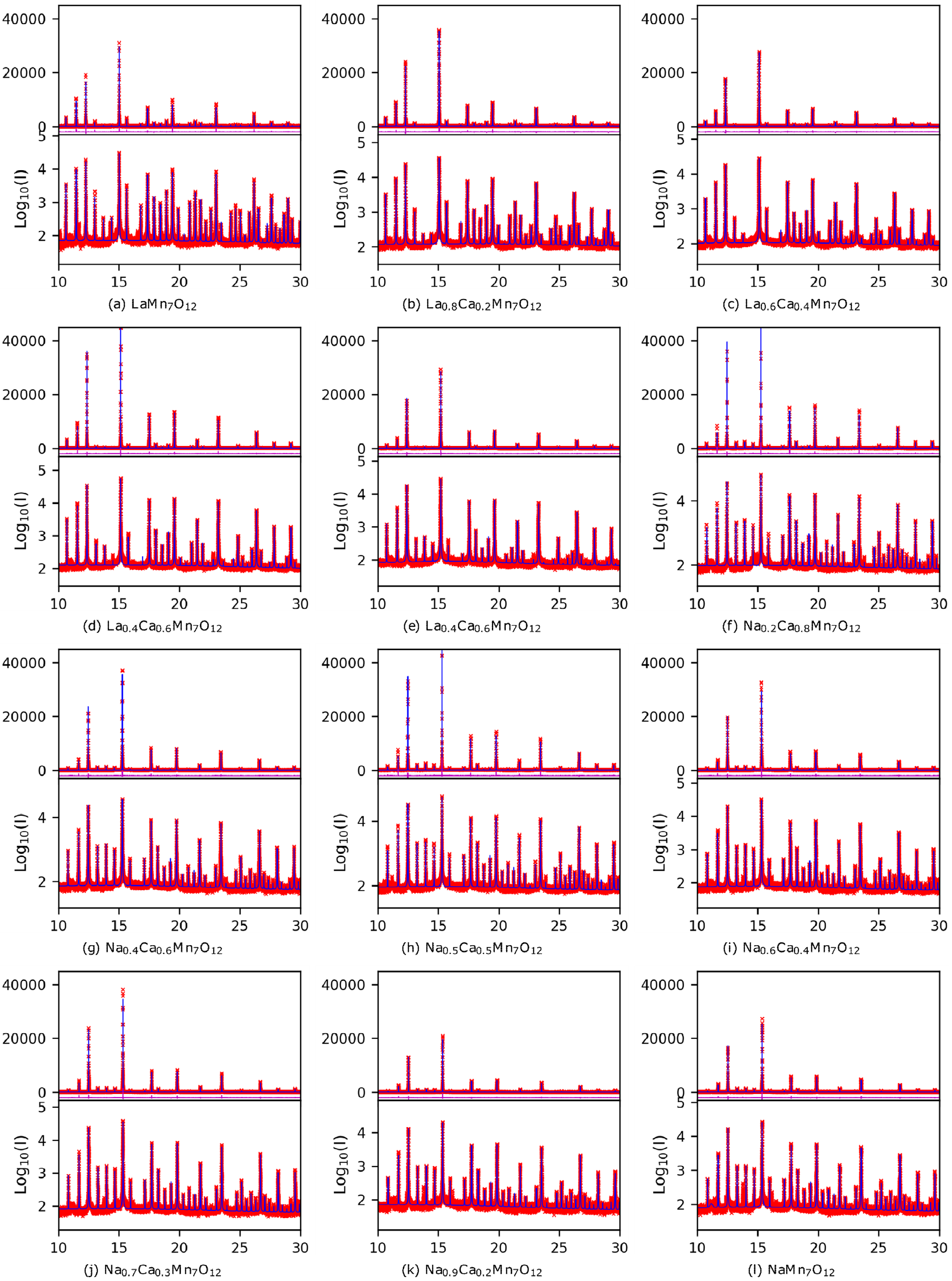

Fig. S1. Representative Riveted fit to ID22 powder x-ray diffraction patterns of (Na/Ca/La)Mn7O12 series at 700 K. Actual fits were performed in the range 2θ = 2.8 to 50 degrees. From these fits the high temperature BVS and octahedral tilt angles are derived that are shown in Table S1 and plotted in Figure 1 of the manuscript. In all instances the same 2θ range in degrees is plotted (wavelength λ = 0.3999 Å).

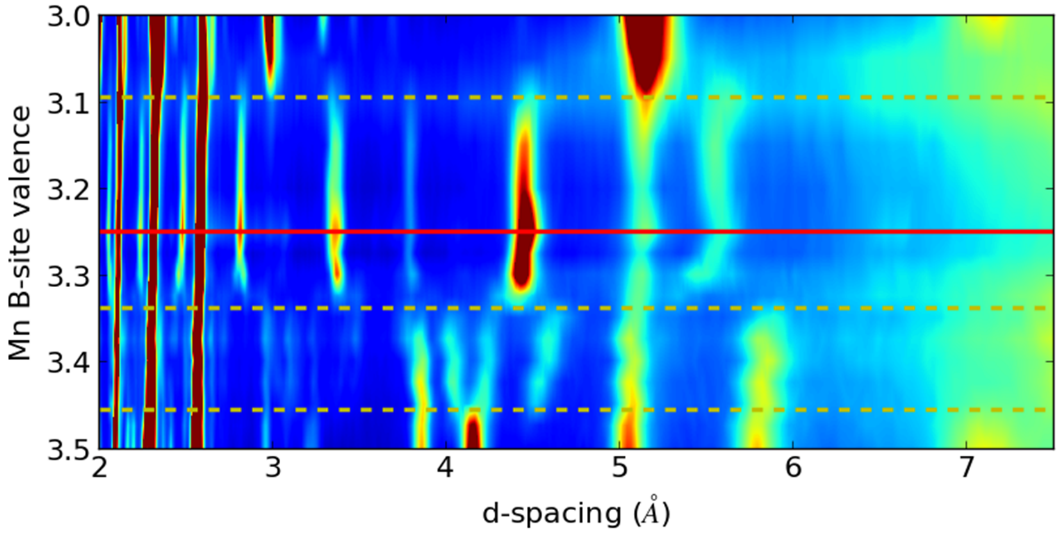

Figure S2: Heatmap view of neutron powder diffraction patterns showing the nuclear and magnetic scattering of (Na/Ca/La)Mn_7O_{12} series. The figure is generated from 16 Neutron diffraction patterns collected at 4 K on the instrument Wombat, ANSTO with λ ≈ 2.41 Å. A linear interpolation is used to generate the pixels for the figure. Four distinct regions of magnetic ordering are evident (horizontal dashed lines) corresponding with the 4 distinct OO structures discussed in the manuscript. The central composition A = Ca, $Mn_B^{3.25+}$ is marked by a solid horizontal red line.

A=	GoF	R_{wp}	Impurities	Notes
Na	1.4	11.73	$Na(Mn_2O_4)$ 4.2%; Bixbyite Mn_2O_3 1.1%	2nd phases with WP refined to 20.6%; UISO constrained to scale as in $Na_{0.8}$
$Na_{0.9}$	0.96	8.83	$Na(Mn_2O_4)$ 1.5%; Bixbyite Mn_2O_3 0.8%	2nd phases with WP = 700 K fit; UISO constrained to scale as in $Na_{0.8}$
$Na_{0.8}$	1.57	11.46	$Na(Mn_2O_4)$ 3.83%; Bixbyite Mn_2O_3 0.3%; HP Mn_3O_4 0.41%	2nd phase not necessary for good fit.
$Na_{0.7}$	1.54	12.32	$Na(Mn_2O_4)$ 0.9%; Bixbyite Mn_2O_3 0.1%; HP Mn_3O_4 0.5%	2nd phase not necessary for good fit.
$Na_{0.6}$	1.21	10.42	Bixbyite Mn_2O_3 0.6%; HP Mn_3O_4 0.8%	2nd phase not necessary for good fit.
$Na_{0.5}$	1.31	10.20	HP Mn_3O_4 1.7%	2nd phase not necessary for good fit.
$Na_{0.4}$	1.26	10.88	Bixbyite Mn_2O_3 0.1%; HP Mn_3O_4 0.4%	2nd phase not necessary for good fit.
$Na_{0.3}$	1.25	11.15	HP Mn_3O_4 1.4%; Bixbyite Mn_2O_3 0.33	2nd phases with WP = 700 K fit; majority phase R-3, monoclinic minority.
$Na_{0.2}$	1.29	11.09	HP Mn_3O_4 1.9%	2nd phase not necessary for good fit.
$Na_{0.1}$	1.59	12.96	HP Mn_3O_4 1.8%	2nd phase not necessary for good fit.
$La_{0.2}$	1.44	12.93	Bixbyite Mn_2O_3 1.1%	2nd phase not necessary for good fit.
$La_{0.4}$	1.42	10.59	Bixbyite Mn_2O_3 0.3%	2nd phase not necessary for good fit.
$La_{0.6}$	1.57	11.42	Bixbyite Mn_2O_3 0.33%	2nd phases with WP = 700 K fit; Majority phase R-3, monoclinic minority.
$La_{0.8}$	1.56	9.98	HP Mn_3O_4 1.4%	2nd phases with WP = 700 K fit
La	1.50	12.44	HP Mn_3O_4 4.0 %	2nd phases with WP = 700 K fit, BISO(La) constrained to = BISO(Mn_A). A/A' peak between 2θ = 8.47 - 8.87° excluded and anti-site order modeled as before.

Table S2: Details of refinements at 80 K against ID22 data. Refined structures are deposited in CIF format as part of the electronic SI. Instrument peak shape was modeled by single fixed Lorenzian contribution. The following parameters are refined for all models. Zero point error; 8 terms of a shifted Chebyshev polynomial + 3 parameters describing a broad Gaussian feature centered at 2θ ~ 5.7° and FWHM of ~ 2.6 ° to model the background; 4 isotropic thermal displacement parameters (for A, A', B and O) unless otherwise indicate in the table. In the case of monoclinic phases, symmetry adapted displacements transforming M1+(a,b,c), M2+(a,b,c), M3+(0,a,0) and M4+ (0,a,0) along with Σ_2(0,0;0,0;a,0;0,0;0,0;0,0) at the Na rich end ($Mn_B^{3.35\text{-}3.5+}$).

At the La rich end of the phase diagram ($Mn_B^{3-3.1+}$), the atomic displacement transforming as Σ_2 are not refined as there are no associated visible superstructure peaks, but secondary displacements transforming as M5+(0,a;0,0;b,0) are refined, their addition justified based on the larger amplitudes of the primary OP M3+ that couple to these. For the central part of the phase diagram with R-3 symmetry ($Mn_B^{3.15-3.325+}$), displacements transforming as M1+(a,a,a), M2+(a,a,a), M3+(a,a,a) and M4+ (a,a,a) and M5+(a,b;a,b;a,b) are refined. An additional phase with identical fractional coordinates and phase fraction consistent with that observations in the cubic data at 700 K are fitted where necessary, as indicated in the table. The microstrain (peak broadening) model used in the main phase of the Na rich end ($Mn_B^{3.35-3.5+}$) and in A = $La_{0.8}Ca_{0.2}$ is the Stephens anisotropic Monoclinic model (9 parameters). A= $La_{0.6}Ca_{0.4}$ uses a Stephens trigonal anisotropic strain model (5 parameters). R-3 phases, A= $La_{0.4}Ca_{0.6}$ - $Na_{0.3}Ca_{0.7}$ uses an isotropic strain model with Gaussian and Lorentzian contributions and Gaussian size broadening model (870 nm). Small levels of impurities are fitted (down to a weight percentage of 0.3%) to prevent any systematic errors when fitting the weak superstructure peaks. Representative fits are given in Fig S3 and S4. Refined parameters for A = $Na_{0.4}Ca_{0.6}$ to Na ($Mn_B^{3.35-3.5+}$) are summarized in Fig S5. Crystallographic information files are included as part of the electronic Supplemental Information.

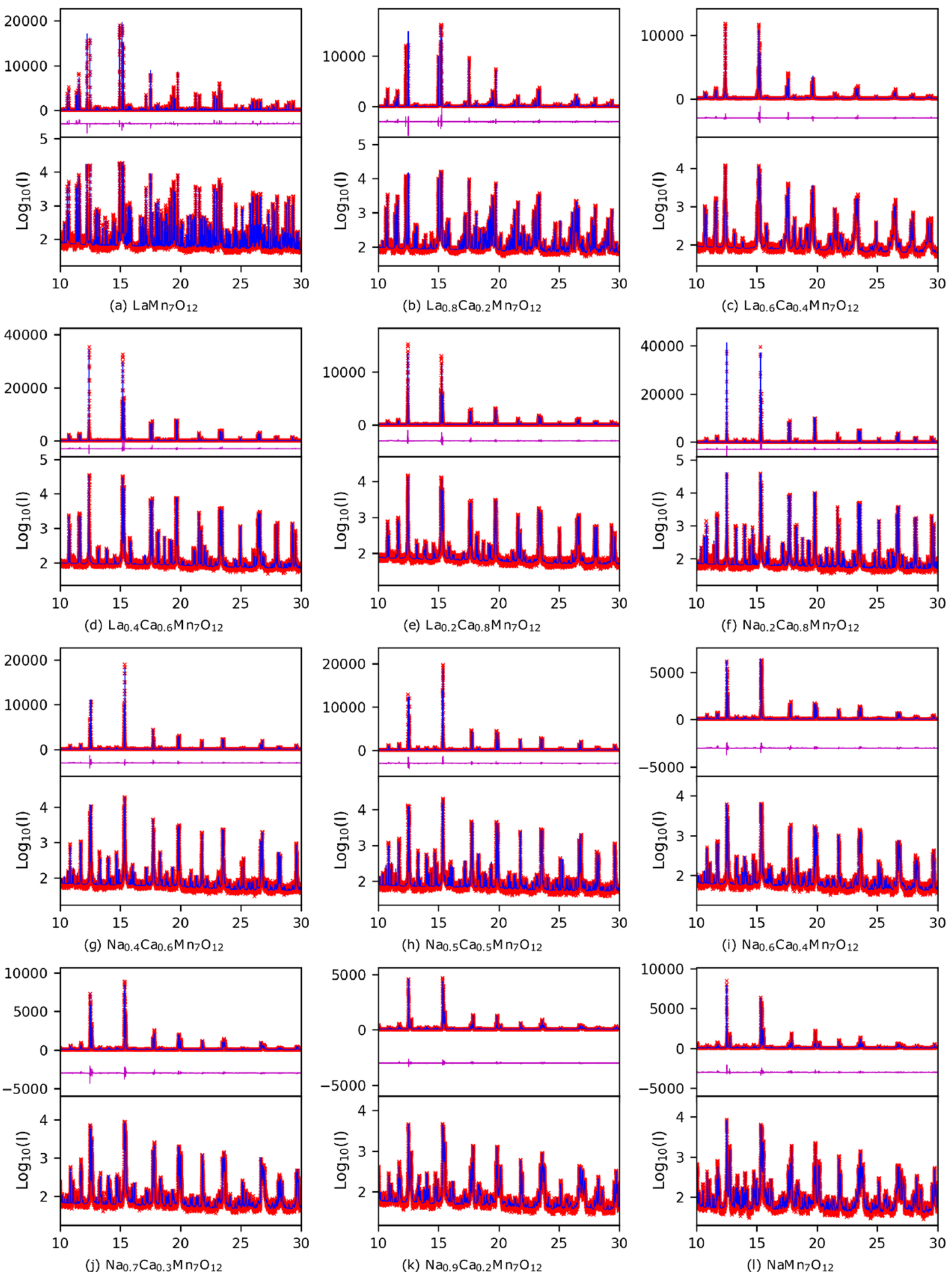

Fig S3. Rietveld fits against 80K ID22 data (λ = 0.39990 Å). Data is in red, model in blue and difference curve in purple. The x-axis scale is 2θ in degrees (°). The actual Rietveld fits were performed in the range 2θ = 2.8 - 40°. The top plot is on a linear scale and the bottom plot on a log scale in each instances. Fits to the very weak superstructure peaks that determine the OO-CD model are shown in Fig S4.

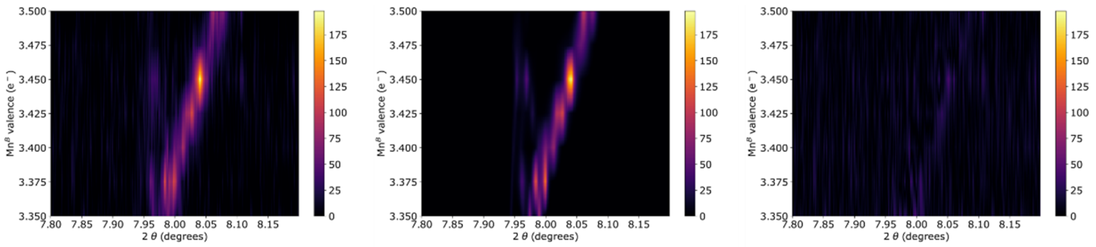

(b) hkl = ($\frac{1}{2}$ 2 $\frac{3}{2}$) / ($\frac{5}{2}$ 0 $\frac{1}{2}$)

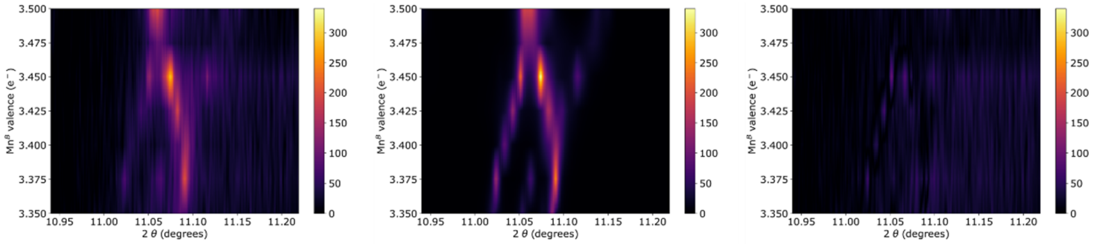

(e) hkl = ($\frac{1}{2}$ 0 $\frac{7}{2}$) / ($\frac{5}{2}$ 2 $\frac{3}{2}$)

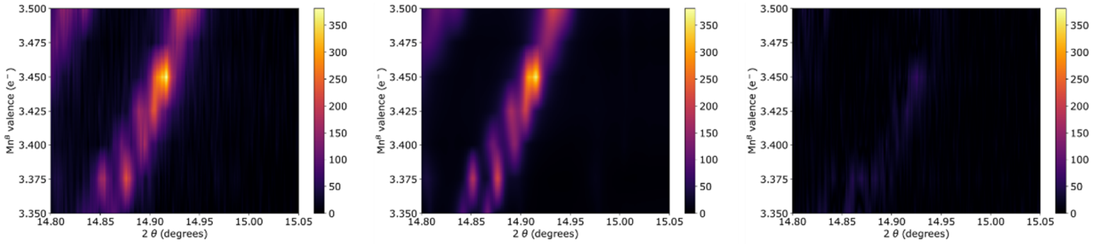

(h) hkl = ($\frac{5}{2}$ 2 $\frac{7}{2}$) / ($\frac{1}{2}$ 4 $\frac{5}{2}$)

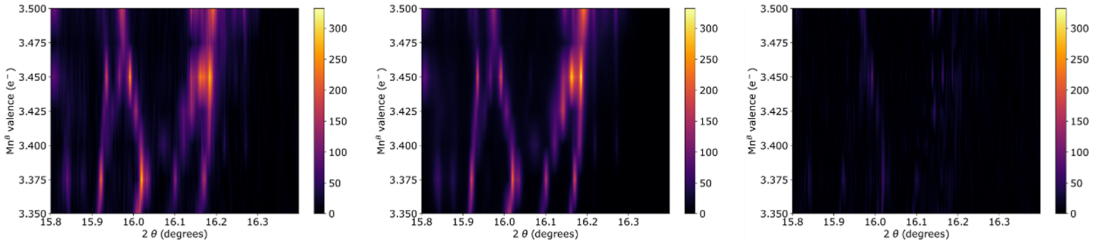

(k) hkl = (1 0 5) / ($\frac{3}{2}$ 2 $\frac{9}{2}$)

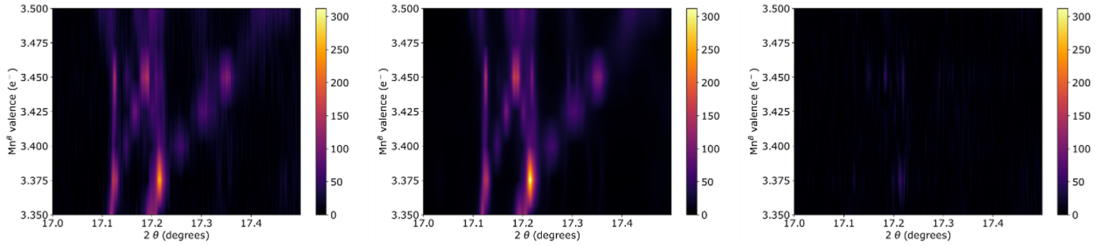

(n) hkl = (2 1 5) / (1 5 2) / (3 1 $\frac{9}{2}$)

Fig S4. Rietveld fits to superstructure peaks by the striped OO-CD model for compositions A = $Na_{0.4}Ca_{0.6}$ ($Mn_B^{+3.35}$) to A = Na ($Mn_B^{+3.5}$) visualized as heat maps. Images for these plots are generated from a linear interpolation between 7 data sets and Rietveld fits at 80 K. Data (left), model (center) and absolute difference (right) are all plotted on the same intensity scale. The *hkls* associated with the intensities

are shown below the plots indexed relative to the parent Im-3 setting. All *hkls* indices should be divide by 2 to recover an indexation relative to the smaller conventional *Pm-3m* perovskite unit cell.

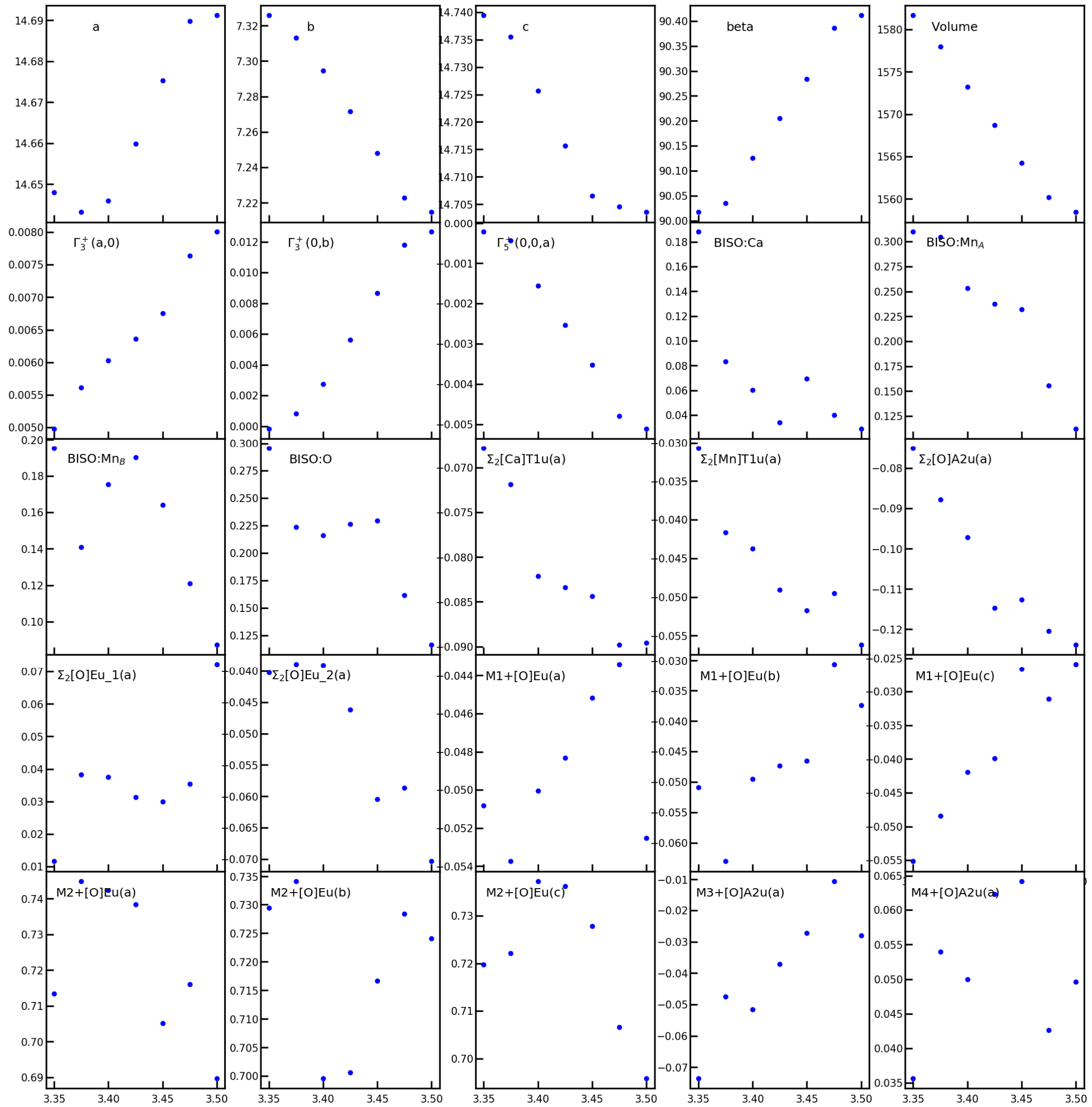

Fig. S5. Parameters extracted from Rietveld refinements of A = $Na_{0.4}Ca_{0.6}$ ($Mn_B^{+3.35}$) to A = Na ($Mn_B^{+3.5}$) against the ID22 high resolution diffraction data at 80 K. Mode amplitudes are A_p values as defined in *ISODISTORT* normalized to the *Pm-3m* perovskite unit cell. Lattice parameters, isotropic displacement parameters (BISO) and most symmetry adapted displacement parameters evolve continuously across the series (or change little) indicating that the refinements are well behaved. In the refinements, the charge ordering mode M_4^+ flipped sign in A = $Na_{0.4}Ca_{0.6}$ with respect to the rest of the series, and so has been constrained here. The pseudo symmetry at this doping level essentially makes it impossible to determine the phase of this with respect with respect to the M_3^+ and SM2 OO.

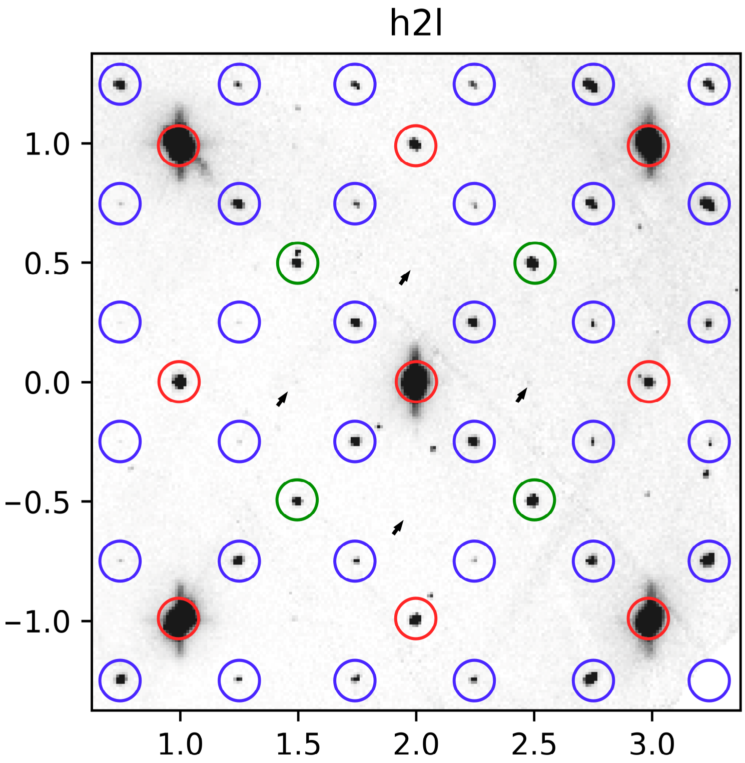

Fig. S6 Reciprocal space reconstructions from oversaturated images collected on A = $Na_{0.4}Ca_{0.6}$ on I19, Diamond Light Source, on a micron-sized grain with 6 twin domains. The *h2l* plane is indexed with respect to the cubic *Pm-3m* perovskite aristotype. Reflections allowed in the aristotype (red), due propagation vectors k = [½ 0 ½] (green) that correspond with cation order (M_1^+), octahedral rotation (M_2^+(a;b;c)) and JT distortions (M_3^+ (0;a;0)) and k = [¼ 0 ¼] (blue) corresponding with JT distortions (Σ_2) are indicated by circles. The absence of intensity at k = [½ 0 0], indicated with arrows, illustrates that only one arm of the star of k = [¼ 0 ¼] is active. The absence of diffuse scattering suggest that there is no correlated disorder associated with the orbital-ordered and charge-disordered layers of the model presented in the manuscript.

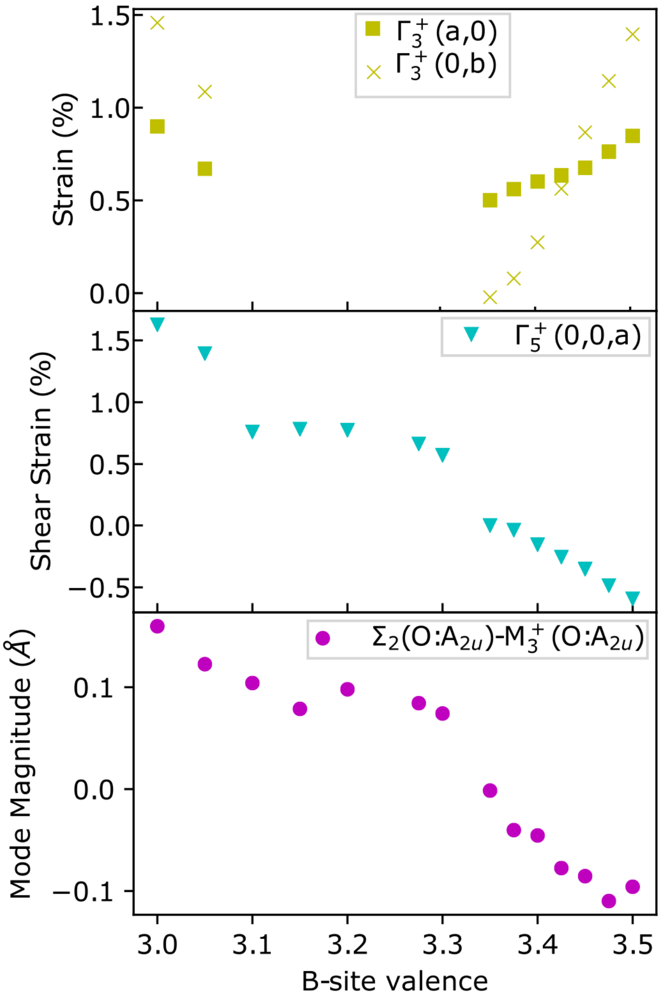

Figure S7: Macrostrain from A = $La_{1-x}Ca_x$ and A = $Na_{1-x}Ca_x$ plotted as a function of Mn_B^{n+} at 80 K. Between n = 3.1 and 3.325 a rhombohedral phase is adopted which does not have any symmetry breaking strains transforming as Γ_3^+. For the other phases, region in which Γ_3^+(0,b) tend to zero (n =3.35+) or Γ_3^+(0,b) = √3Γ_3^+(a,0) (n =3.0 and 3.5+), correspond to metrically pseudo tetragonal distortions. Across the entire phase diagram, the shear strain (transforming as Γ_5^+) correlates strongly with the difference in mode amplitudes between the Σ and M-point orbital ordering modes, indicating that it is a cancelation, in the Landau-style free energy expansion about the high symmetry cubic phase, that leads to zero shear strain around n = 3.35 - 3.375.

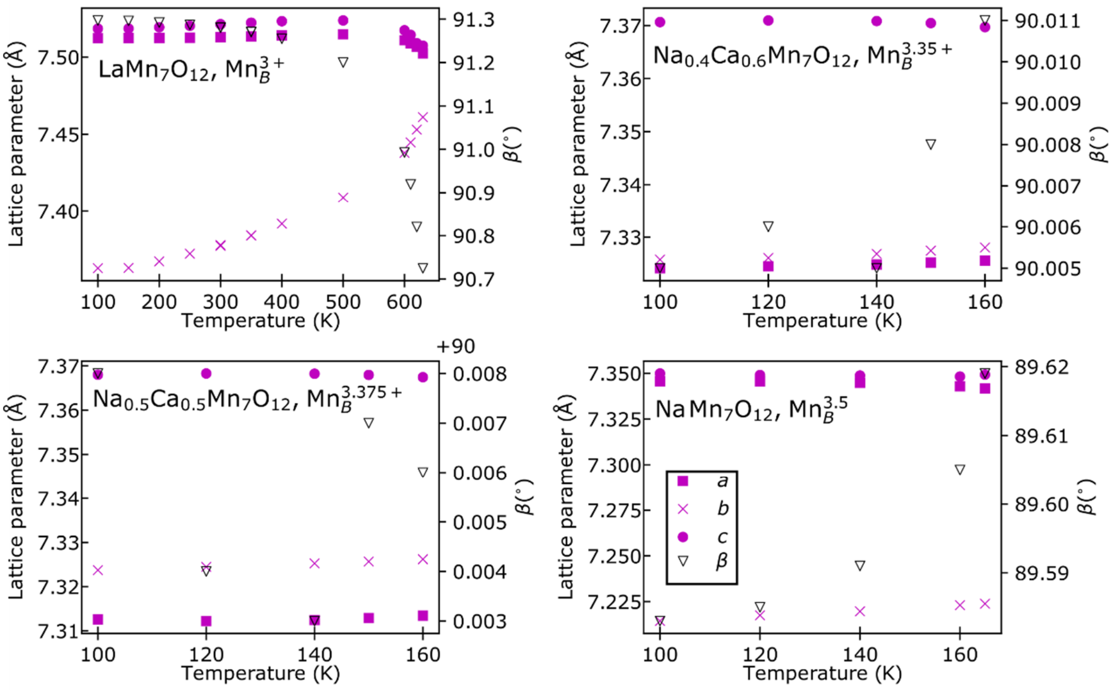

Figure S8: Variable temperature lattice parameters for pseudo tetragonal OO phases extracted from Rietveld refinement against SXRD patterns obtained at beamline 09A, Taiwan Photon Source with 15 keV beam energy. The temperature dependence of the lattice parameters shows that the pseudo symmetry doesn't simply occur accidental at one temperature but is maintained below the OO-CD phase transition, indicating that it is intrinsically linked to the underlying electronic order. We note that for A=$Na_{0.4}Ca_{0.6}$ and $Na_{0.5}Ca_{0.5}$, the refined variation in β, which is of the order of 6 thousands of a degree across the temperature range, is likely just experimental noise. A better appreciation of the intrinsic resolution of the current experiment can be gained from Fig S9 that established the sheer monoclinic (Γ_5^+) microstrain as being no larger than the microstrain in the samples, and hence we refer to these phases as metrically pseudo-tetragonal.

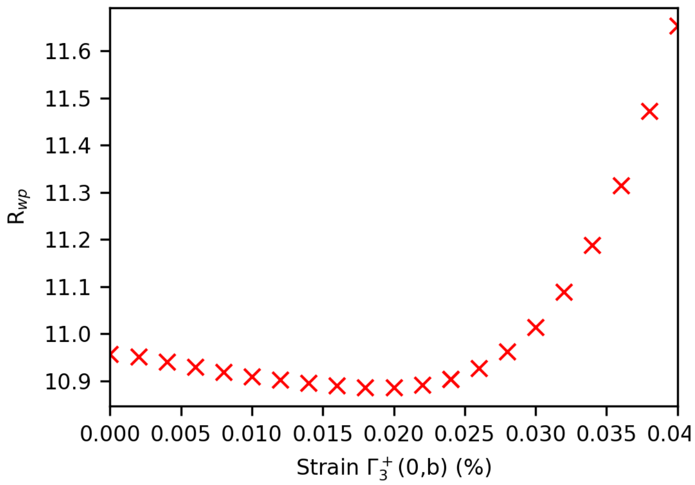

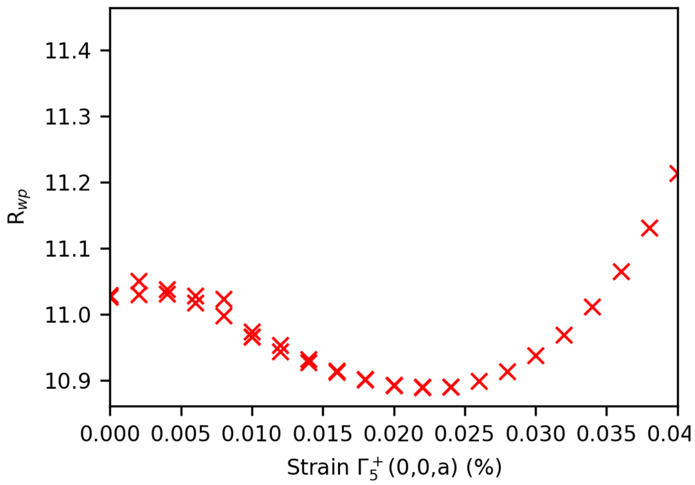

Fig. S9. Macrostrain (lattice parameter) resolution for pseudo tetragonal $Na_{0.4}Ca_{0.6}$-Mn_7O_{12} ($Mn_B^{+3.335}$) at 80 K in the OO-CD state. Plots evidences that lattice parameters are pseudo tetragonal within ~0.02%. Each point on the graphs represents a refinement in which the orthorhombic (transforming as Γ_3^+(0,b)) or sheer monoclinic (Γ_5^+) type strain (w.r.t. the parent setting) has been fixed and a Rietveld refinement performed optimizing all other parameters, including anisotropic peak broadening parameters. The minimum in the macrostrain for Γ_3^+(0,b) and Γ_5^+ comes in both cases near 0.02% approximately equal to the macrostrain (e_0 = 0.016%) of the sample that effectively determines the resolution of the current experiment. By contrast, the pure tetragonal strain transforming as Γ_3^+(a,0) if about 25 time higher than this (0.5%) and produces clearly resolvable peak splitting (see Fig. S10).

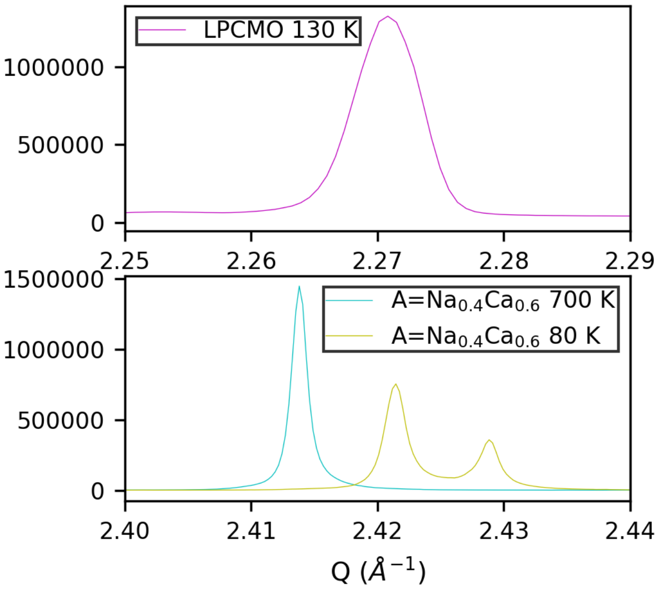

Fig S10. Comparison of micro- and macrostrain using the reflection equivalent to hkl = (1 0 1) in the cubic Pm-3m perovskite structure of the prototype system A = $Na_{0.4}Ca_{0.6}$ at 700 and 80 K of LPCMO at 130 K. Microstrain is 3-4 times higher in LPCMO (0.04 %) , while the orthorhombic type macrostrain (0.18%) is 3 times lower than in A = $Na_{0.4}Ca_{0.6}$ at 80 K (0.56%, evident by the split peak). This simultaneous reduction in macrostrain and increase in microstrain has the effect of producing a single peak for LPCMO with ~6 larger FWHM than in the case of A = $Na_{0.4}Ca_{0.6}$.

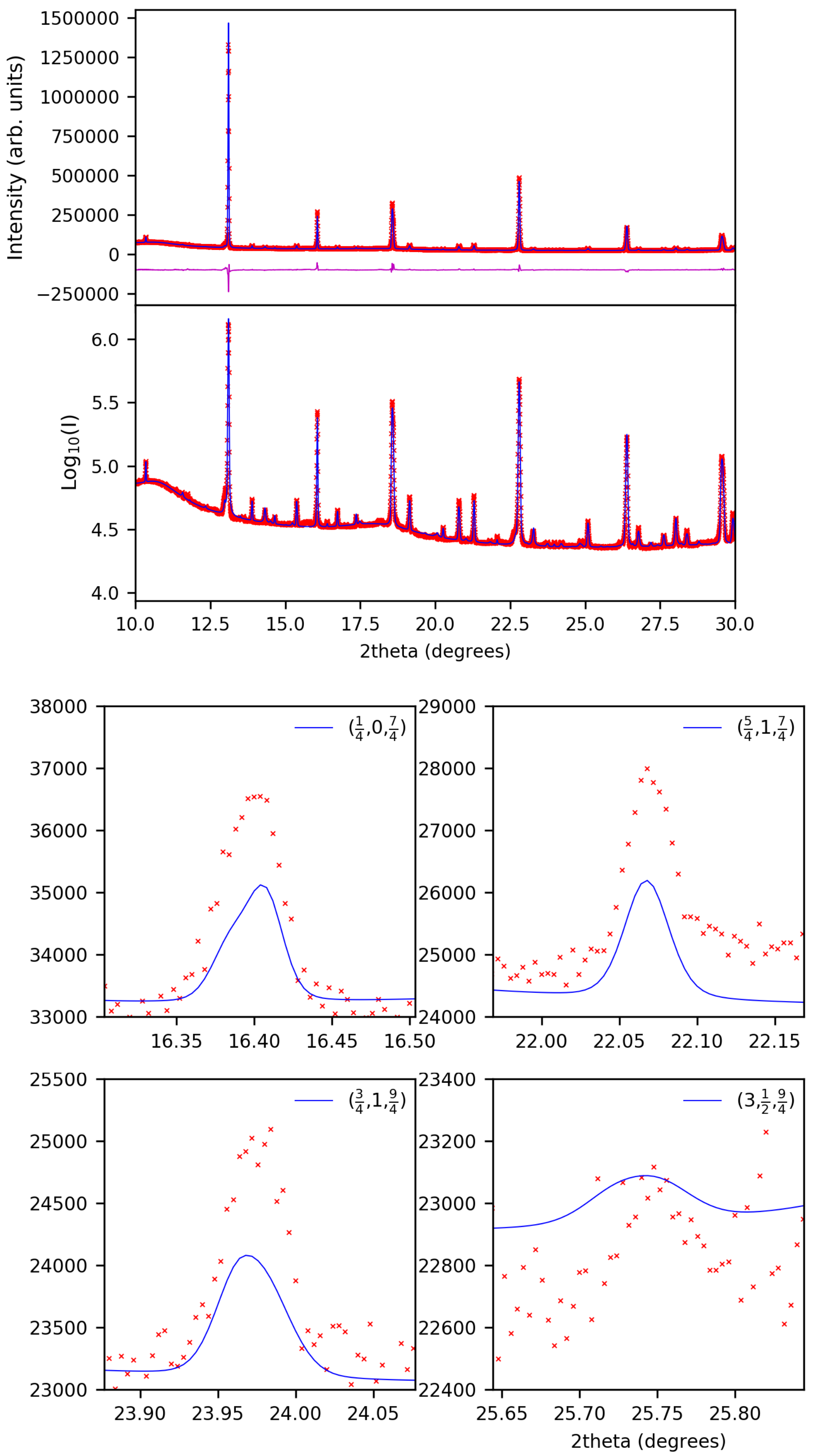

Fig S11. OO-CD model plotted against LPCMO data collected at beamline 09A at Taiwan Photon Source at 130 K. The superstructure peaks are weaker than in the prototype system on account of both the increased microstrain peak broadening and phase coexistence between insulating (OO) and metallic phases, meaning that the

parameters cannot be freely refined due to an insufficient number of superstructure peaks being observable above the background level. However, the data is consistent with the OO-CD model derived against the prototype systems at the equivalent doping value (3/8th , $Mn_B^{3.375+}$) . The peaks are indexed with respect to the pseudo cubic *Pm-3m* aristotype with *a* = 3.844 Å. Equivalent reflections to those plots in Fig S4 for the prototype system have been given where possible. The present *hkl*s may be convert to the ~2*a* porotype setting (Im-3) by multiply the indices by 2.

		Mode amplitudes A_p (Å) of model indicated						
	Mode	$Na_{0.5}$	$Na_{0.4}$	DFT (AFM) U = 0.5 e J = 0	DFT(FM) U = 0.5 e, J-0	LPCMO	PMO DFT(AFM)	PMO DFT(FM)
SM2	[A:a:dsp]T1u(a)	-0.0675	-0.0637	-0.0324	-	-0.03688	-0.0323	-0.0011
	[Mn:b:dsp]T1u(a)	-0.0392	-0.0289	-0.0233	-	-0.02079	-0.0318	-0.0009
	[O:c:dsp]A2u(a)	-0.0830	-0.0706	-0.0626	-	-0.04320	-0.0670	-0.0011
	[O:c:dsp]Eu_1(a)	0.0359	0.0104	0.0276	-	0.01965	0.0619	0.0012
	[O:c:dsp]Eu_2(a)	-0.0363	-0.0378	-0.0255	-	-0.01766	-0.0331	-0.0005
M1+ (a;b;c)	[O:c:dsp]Eu(a)	-0.0503	-0.0476	-0.0547	-0.0612	0	0.0047	0.0002
	[O:c:dsp]Eu(b)	-0.0592	-0.0477	-0.0630	-0.0615	-	-	-
	[O:c:dsp]Eu(c)	-0.0452	-0.0518	-0.0588	-0.0556	-	-	-
M2+ (a;b;c)	[O:c:dsp]Eu(a)	0.7000	0.6703	0.6748	0.6774	0.37760	0.2491	0.2301
	[O:c:dsp]Eu(b)	0.6896	0.6850	0.6838	0.6824	-	-	-
	[O:c:dsp]Eu(c)	0.6782	0.6757	0.6811	0.6787	-	-	-
M3+ (0;a;0)	[O:c:dsp]A2u(a)	0.0446	0.0685	0.0345	0.0001	0.03453	0.0357	0.0042
M4+ (0;a;0)	[O:c:dsp]A2u(a)	-0.0509	-0.0332	-0.0401	0.0001	-	-0.0346	-0.0003
M5+(0,a;0,0;b,0)	[O:c:dsp]Eu(a)	0	0	-0.0305	0.0002	-	-	-
	[O:c:dsp]Eu(b)	0	0	0.0246	0.00000	-	-	-
S1	[A:a:dsp]T1u(a)	0	0	0.0086	-	0	0.0007	0.0016
	[Mn:b:dsp]T1u(a)	0	0	-0.0080	-	0	0.0010	0.0011
	[O:c:dsp]Eu_1(a)	0	0	0.0023	-	0	0.0028	-0.0020
	[O:c:dsp]Eu_2(a)	0	0	-0.0086	-	0	0.0004	0.0019
S2	[A:a:dsp]T1u(a)	0	0	0.0084	-	0	0.0152	0.0003
	[O:c:dsp]Eu_1(a)	0	0	-0.0072	-	0	-0.0119	-0.0025
	[O:c:dsp]Eu_2(a)	0	0	-0.0027	-	0	-0.0101	-0.0027
R5-	[O:c:dsp]Eu(a)	-	-	-	-	-0.38782	0.3888	0.3579
	[O:c:dsp]Eu(b)	-	-	-	-	0.38782	0.3441	0.3613
R4-	[A:a:dsp]T1u(a)	-	-	-	-	0.01410	-0.0233	-0.0240
	[A:a:dsp]T1u(b)	-	-	-	-	0.01410	0.0286	0.0239
	[O:c:dsp]Eu(a)	-	-	-	-	0.07655	0.0170	0.0161
	[O:c:dsp]Eu(b)	-	-	-	-	0.07655	-0.0147	-0.0160
X5-	[A:a:dsp]T1u(a)	-	-	-	-	0.10352	0.0935	0.0906
	[A:a:dsp]T1u(b)	-	-	-	-	0.10352	0.1058	0.0915
	O:c:dsp]Eu(a)	-	-	-	-	-0.05626	-0.0343	-0.0330
	O:c:dsp]Eu(b)	-	-	-	-	-0.05626	-0.0185	-0.0320

Table S3. Comparison of experimental versus DFT mode amplitude. Mode amplitudes

are in Å and represent the square root of the sum of the square of all atomic displacements associated with a given mode within the primitive cubic Pm-3m perovskite unit cell (A_p values as defined in ISODISTORT).

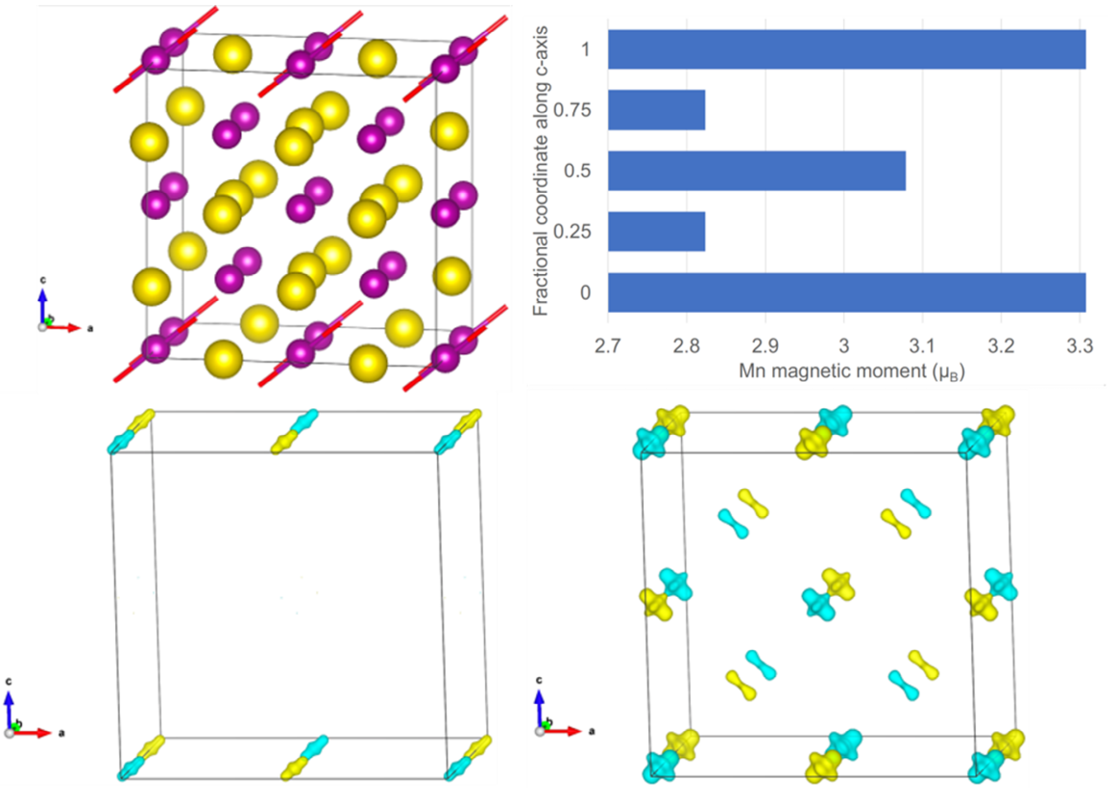

Fig S12 AFM PMO perovskite structure at $Mn_B^{3.375+}$ doping level (top left); Pr yellow, Mn purple, longest Mn-O bond lengths shown (>2.05Ang). Layer-by-layer Mn magnetic moment (top right). Magnetization isosurfaces of states just below (0 to -1eV) Fermi level (bottom). Magnetisation density isosurface values: +-0.123 (left) and +-0.045 (right). (Positive blue, negative yellow)**.**

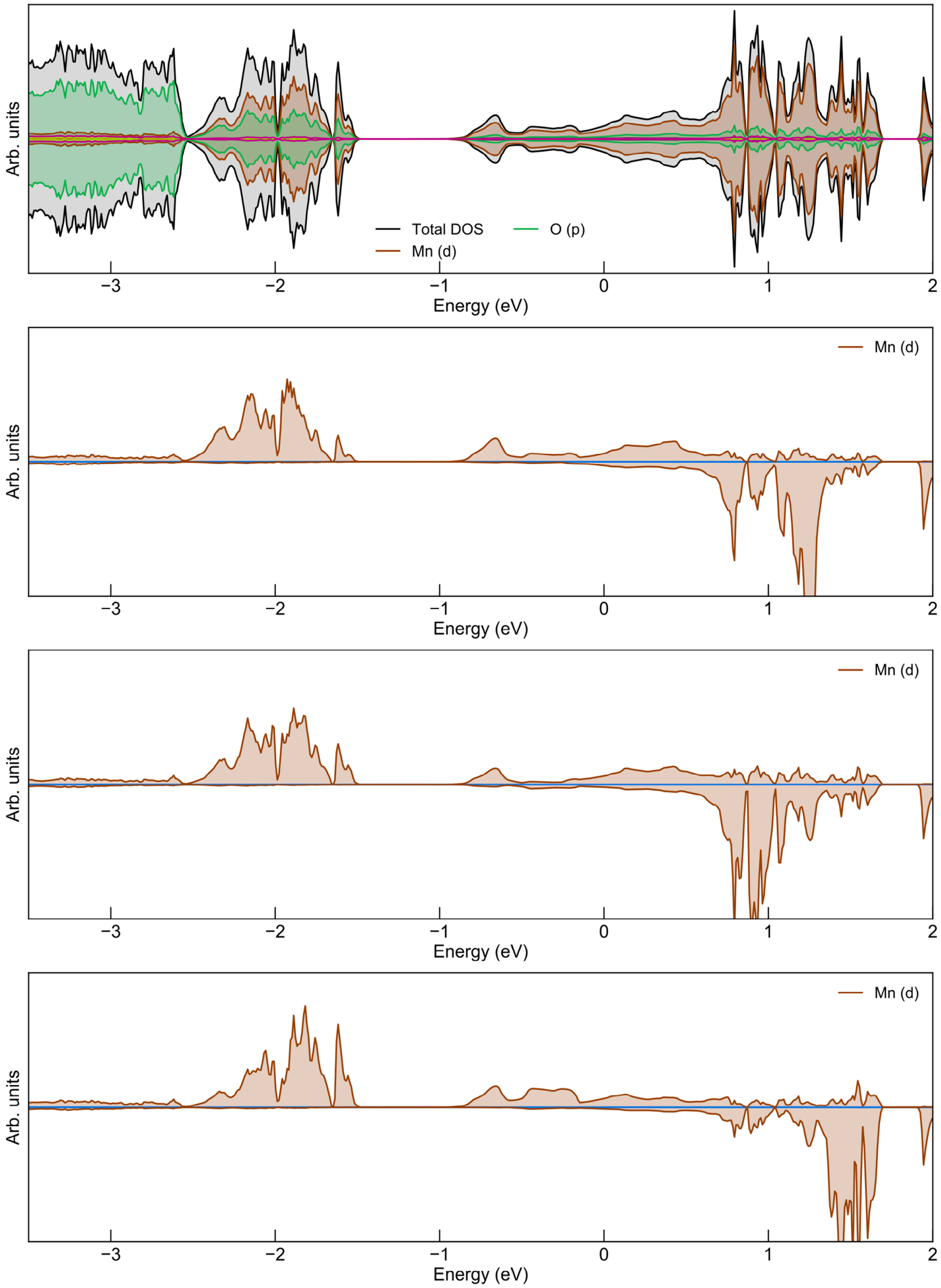

Fig S13. Total density of states of AFM PMO at $Mn_B^{3.375+}$ doping level (top) followed by projected density of states on a spin up Mn site within each layer normal to the c-axis (z=0.5, z=0.25/0.75 and z=0/1, from top to bottom). Mn projected DOS have been scaled compared to the total for clarity. The states just below the fermi level are predominantly of z=0 Mn d-character, in agreement with the spin isosurface plots in fig. S15.

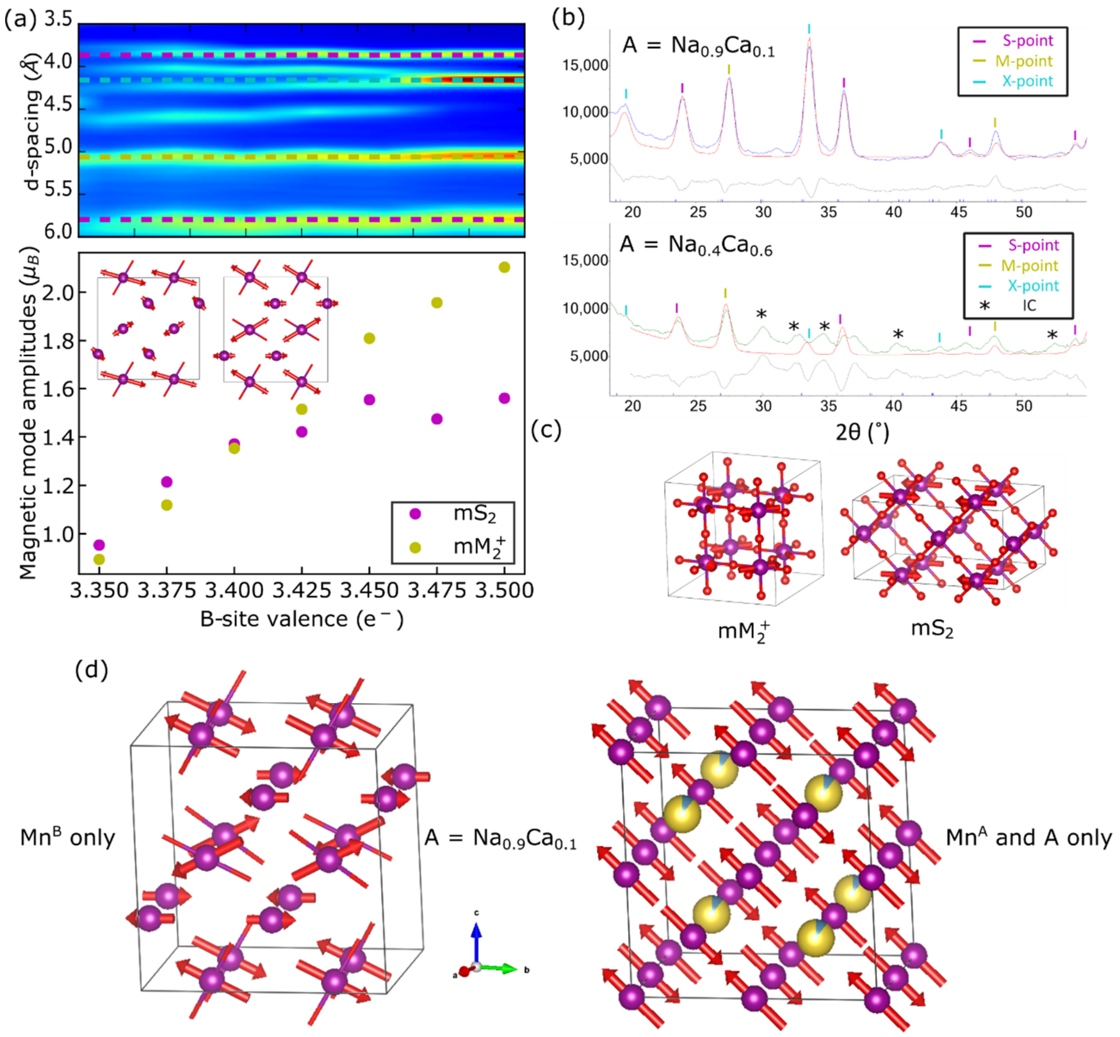

Fig S14. (a) Heat map showing the evolution of the neutron magnetic scattering and associated symmetry adapted magnetic modes for Mn B-site as a function of formal valence. The order parameter directions associated with the magnetic modes, as defined in ISODISTORT are mM2+(a;0;a) and mS2(0,b;0,0;0,0;0,0;0,0;0,0) Magnetic modes are normalized such that the amplitudes correspond to the average moment per Mn^B (primitive Pm-3m unit cell). The commensurate part of the magnetic structure in the **bc** plane is visualized in the inset where the interactions along the out-of-plane **a** direction are AFM. The amplitudes of the magnetic moments are extracted via symmetry adapted refinement in Topas against data collect on Wombat, ANSTO (λ ≈ 2.4 Å) at 4K. Representative Rietveld fits are shown in (b) from which the magnetic mode amplitudes are extracted that are plotted in (a). The 2θ range plotted corresponds approximately to the d-spacing range 7.3 – 2.6 Å. Diffraction due to the ordering of the magnetic moments on Mn^A can also be seen transforming as mX3+(0;0;a)mX5+ (a,0;0,a;0,0) with propagation vectors k = [½ 0 0] + [0 ½ 0] + [0 0 ½] (cyan in (a) and (b)), but this ordering is only shown in the right hand part of (d) where and enlarged view of the B-site magnetic sublattice is also

given with the JT long axes indicated by stick bonds. Intensity due to the evolution of the incommensurate component of the magnetic structure can be seen at around d-spacing = 4.6 Å in (a) and is marked with * in the Rietveld fits. The intensity associated with this increasing towards the 3/8th doping level ($Na_{0.4}Ca_{0.6}$).

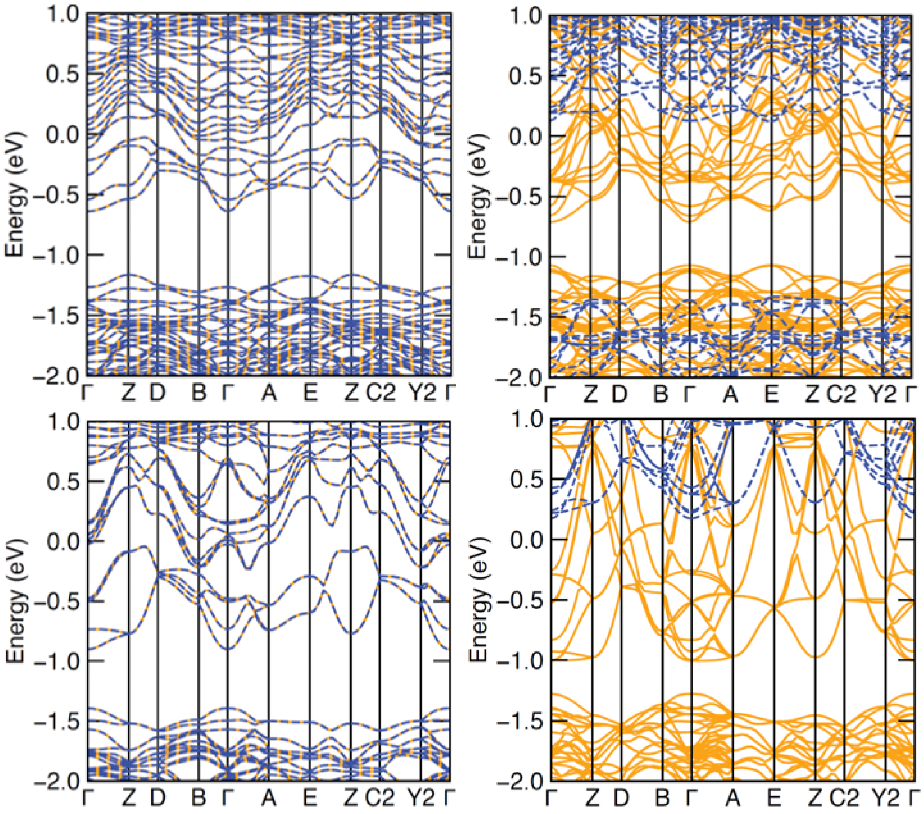

Fig S15 Band structure for 134 Prototype system (top) and PMO structure (bottom) at $Mn_B^{3.375+}$ doping level. In each case the band structure from the AFM relaxed structure is shown left and for FM right.
