# Supplementary material for: Striping of orbital-order with charge-disorder in optimally doped manganites
Source: Nat Commun. 2021 Nov 3;12:6319. doi: 10.1038/s41467-021-26625-w (PMC8566459; doi:10.1038/s41467-021-26625-w)
Supplement: Supplementary file 3 — Description of Additional Supplementary Files. [file 41467_2021_26625_MOESM3_ESM.pdf]

Description of Additional Supplementary Files

File Name: Supplementary Data 1

Description:

Crystallographic Information Files output from Rietveld refinements against high resolution synchrotron powder diffraction data collected at 80 and 700 K. The refinement procedures are as described in the Methods section and detail in the Supplementary Information PDF file in Table S1 and S2 and Fig. S5.
